# *Lycium barbarum* Polysaccharides Reduce Exercise-Induced Oxidative Stress

**DOI:** 10.3390/ijms12021081

**Published:** 2011-02-09

**Authors:** Xiaozhong Shan, Junlai Zhou, Tao Ma, Qiongxia Chai

**Affiliations:** 1 Department of Physical Education and Military Training, Zhejiang University of Technology, Hangzhou, Zhejiang Province 310014, China; E-Mails: zjlzjut@126.com (J.Z.); MaTao2005@foxmail.com (T.M.); 2 Ningbo Lihuili Hospital, Ningbo, Zhejiang Province 315041, China; E-Mail: chaiqiongxia@163.com (Q.C.)

**Keywords:** *Lycium barbarum* polysaccharides, exercise, oxidative

## Abstract

The purpose of the present study was to investigate the effects of *Lycium barbarum* polysaccharides (LBP) on exercise-induced oxidative stress in rats. Rats were divided into four groups, *i.e*., one control group and three LBP treated groups. The animals received an oral administration of physiological saline or LBP (100, 200 and 400 mg/kg body weight) for 28 days. On the day of the exercise test, rats were required to run to exhaustion on the treadmill. Body weight, endurance time, malondialdehyde (MDA), super oxide dismutase (SOD) and glutathione peroxidase (GPX) level of rats were measured. The results showed that the body weight of rats in LBP treated groups were not significantly different from that in the normal control group before and after the experiment (*P* > 0.05). After exhaustive exercise, the mean endurance time of treadmill running to exhaustion of rats in LBP treated groups were significantly prolonged compared with that in the normal control group. MDA levels of rats in LBP treated groups were significantly decreased compared with that in the normal control group (*P* < 0.05). SOD and GPX levels of rats in LBP treated groups were significantly increased compared with that in the normal control group (*P* < 0.05). Together, these results indicate that LBP was effective in preventing oxidative stress after exhaustive exercise.

## 1. Introduction

*Lycium barbarum* belongs to the plant family Solanaceae. Red-colored fruits of *Lycium barbarum*, also called *Fructus lycii* or *Gouqizi*, have been used as a traditional Chinese herbal medicine for thousands of years [1]. In traditional Chinese medicine literature, it has been known for balancing “Yin” and “Yang” in the body, nourishing the liver and kidney and improving visual acuity [2,3]. *Lycium barbarum* fruits have a large variety of biological activities and pharmacological functions and play an important role in preventing and treating various chronic diseases, such as diabetes, hyperlipidemia, cancer, hepatitis, hypo-immunity function, thrombosis, and male infertility [4–7]. In fact, in 1983 the Ministry of the Public Health of China approved *Lycium barbarum* fruits to be marketed as a botanical medicine. Various chemical constituents are found in *Lycium barbarum* fruits. The polysaccharides isolated from the aqueous extracts of *Lycium barbarum* have been identified as one of the active ingredients responsible for the biological activities [7,8]. Previous studies have shown that *Lycium barbarum* polysaccharides (LBP) can enhance exercise endurance capacity, reduce fatigue and exhibit antioxidant activity *in vitro* and *in vivo* [9–13].

Regular physical exercise has many health benefits including a lowered threat of all-cause mortality along with a reduced risk of cardiovascular disease, cancer, and diabetes [14–16]. However, strenuous physical exercise with dramatically increased oxygen uptake is associated with the generation of free radicals and reactive oxygen species (ROS), such as superoxide and hydrogen peroxide, which might cause lipid peroxidation of polyunsaturated fatty acids in membranes, DNA damage, and decreases antioxidant levels in target tissues and blood [17–19]. Oxidative stress can be defined as an imbalance between oxidative reactions and antioxidant capacity that results directly or indirectly in cellular damage [20]. During the past three decades, our knowledge about the biological implications of exercise-induced oxidative stress has expanded rapidly.

Antioxidants are substances that help reduce the severity of oxidative stress either by forming a less active radical or by quenching the reaction. The literature suggests that dietary antioxidants may prevent muscle damage because they are able to detoxify some peroxides by scavenging ROS produced during exercise [21–25]. *Lycium barbarum* polysaccharides (LBP), due to their antioxidant properties, may be applicable in the treatment of disorders in which oxidative stress is involved, including exercise-induced oxidative stress. Therefore, the purpose of this study was to investigate the effects of LBP on exercise-induced oxidation in male rats.

## 2. Results and Discussion

### 2.1. Effects of LBP on Body Weight and Endurance Time of Rats

As shown in Table 1, the body weight of rats in LBP treated groups (low-dose LBP treated (LT), middle-dose LBP treated (MT) and high-dose LBP treated (HT)) were not significantly different from those in the normal control group (NC) before and after the experiment (*P* > 0.05), which means the LBP had no effect on body weight. The mean endurance time of treadmill running to exhaustion of rats in LBP treated groups (LT, MT and HT) were significantly prolonged compared to that in the normal control group (NC) (*P* < 0.05), which was 1.38, 1.45 and 1.55 times that in the NC group, respectively. The results suggested that different doses of LBP might significantly prolong the endurance time, which suggests that LBP might elevate the exercise tolerance of rats.

### 2.2. Effects of LBP on Malondialdehyde (MDA) Level of Rats after Exhaustive Exercise

**Figure 1.**
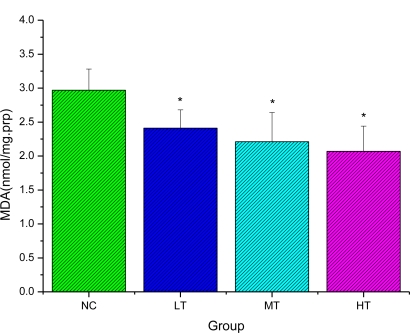
Effects of LBP on malondialdehyde (MDA) level of rats after exhaustive exercise (mean ± SD, n = 12). *p < 0.05 as compared with the normal control group (NC).

Malondialdehyde (MDA) has been the most widely used parameter for evaluating oxidative damage to lipids, although it is known that oxidative damage to amino acids, proteins and DNA also causes release of MDA. Most studies show that endurance exercise causes an increase in MDA [26–28]. As shown in Figure 1, after exhaustive exercise, MDA levels of rats in the LBP treated groups (LT, MT and HT) were significantly decreased compared with those in the normal control group (NC) (*P <* 0.05). The results suggested that different doses of LBP could reduce lipid per-oxidation during exercise.

### 2.3. Effects of LBP on Super Oxide Dismutase (SOD) and Glutathione Peroxidase (GPX) Level of Rats after Exhaustive Exercise

Antioxidant enzymes, which provide the primary defense against ROS generated during exercise, may be activated selectively during an acute bout of strenuous exercise depending on the oxidative stress imposed on the specific tissues as well as the intrinsic antioxidant defense capacity [16,29,30]. Superoxide dismutase reduces superoxide to hydrogen peroxide; and glutathione peroxidase reduces hydrogen peroxide from the SOD reaction to water. In addition, glutathione peroxidase can reduce lipid peroxides directly [31,32]. As shown in Table 2, after exhaustive exercise, SOD and GPX levels of rats in the LBP treated groups (LT, MT and HT) were significantly increased compared with those in the normal control group (NC) (*P* < 0.05). The results indicate that different doses of LBP were able to up-regulate antioxidant enzyme activities to protect against oxidative stress induced by acute exercise. This is probably due to the antioxidant activity of LBP *per se*.

## 3. Experimental Section

### 3.1. Chemicals

Reagent kits for the determination of malondialdehyde (MDA), super oxide dismutase (SOD) and glutathione peroxidase (GPX) were purchased from Jiancheng Biotechnology Co. (Nanjing, China). All other reagents were purchased from either Sigma Chemical Co. (St. Louis, U.S.) or Sinopharm Chemical Reagent Beijing Co., Ltd (Beijing, China).

### 3.2. Plant Materials

The dried *Lycium barbarum* fruits were purchased from Hangzhou city herb market (Zhejiang, China). The plants were identified by Professor Li in the Institute of Zhejiang Institute of Botany, China. A voucher specimen (ZJB-67581) is deposited in the Herbarium of the Zhejiang Institute of Botany.

### 3.3. Preparation of Lycium Barbarum Polysaccharides

*Lycium barbarum* polysaccharides were prepared as described previously [1,33,34]. In brief, 100 g of dried fruit were ground to fine powder and put in 1.5 L of boiling water and decocted for 2 h by a traditional method for Chinese medicinal herbs. The decoction was left to cool at room temperature, filtered and then freeze-dried to obtain crude polysaccharides. The dried crude polysaccharides were refluxed three times to remove lipids with 150 mL of chloroform:methanol solvent (2:1) (v/v). After filtering, the residue was air-dried. The resulting product was extracted three times in 300 mL of hot water (90 °C) and then filtered. The combined filtrate was precipitated using 150 mL of 95% ethanol, 100% ethanol and acetone, respectively. After filtering and centrifugation, the precipitate was collected and vacuum-dried, giving the desired *Lycium barbarum* polysaccharides (LBP). The content of LBP was measured by phenol sulfuric method [35]. Results showed that the content of the polysaccharides in the extract may reach 95.18%.

### 3.4. Animals and Treatments

Eight-week-old male Sprague-Dawley rats, weighing 280 to 300 g, were purchased from Hangzhou animal husbandry center (Zhejiang, China). Rats were maintained on a 12-hour light/dark cycle (lights on 07:00–19:00 hours) in a constant temperature (21–23 °C) and 55 ± 10% relative humidity colony room, with free access to food and water. The approval for this experiment was obtained from the Institutional Animal Ethics Committee of Zhejiang University of Technology (Zhejiang, China). After an adaptation period of a week, 48 rats were randomly divided into four groups, *i.e.*, one control group and three LBP treated groups, of 12 each (Table 3). The volume of administration was 1 mL and the treatments lasted for 28 days. Before the formal experiments, some preliminary experiments were done, and the doses of LBP were determined to be 50 to 600 mg/kg according to relevant literature [36–38]. The results of the preliminary experiments showed that doses of 100 to 400 mg/kg were suitable and effective, with no toxicity in mice. Thus, in this study, the doses of LBP of 100 mg/kg, 200 mg/kg and 400 mg/kg b.w were chosen.

### 3.5. Exercise Protocol

Rats were introduced to treadmill running with 15–20 min exercise bouts at 15–30 m/min for a week to accustom them to running. On the day of the exercise test (the last day of treatment), rats were required to run to exhaustion on the treadmill at a final speed of 30 m/min, 10% gradient and approximately 70–75% VO_2_max (Liu *et al*., 2005). The point of exhaustion was determined when the rat was unable to right itself when placed on its back. The treadmill was provided from Zhishuduobao Biological Technology Company (DB030l device; Beijing, china).

### 3.6. Sample Preparation

All animals were anesthetized with ethyl ether and sacrificed immediately after the exhaustive exercise. Hind-limb skeletal muscle was extracted and frozen in liquid nitrogen for storage at −80 °C until further analysis.

### 3.7. Analytical Oxidative Stress-Associated Parameters

The tissues were homogenized in ice-cold buffer (0.25 M sucrose, 10 mM Tris-HCl, and 0.25 mM phenylmethylsulfonyl fluoride; pH 7.4), and a portion of the homogenate was measured immediately for malondialdehyde (MDA) using a commercial diagnostic kit. Another portion of the homogenate was centrifuged at 10,000 × g for 20 min at 4 °C; super oxide dismutase (SOD) and glutathione peroxidase (GPX) activities in the supernatant were measured using commercial diagnostic kits.

### 3.8. Statistical Analysis

All values are expressed as mean ± standard deviation. Statistical comparisons were made by one-way ANOVA and correlation analysis was performed by Pearson product moment using SPSS version 13.0 (SPSS Inc., Chicago, IL, U.S.). Statistical significance was defined as *P* < 0.05.

## 4. Conclusions

The present results suggest that *Lycium barbarum* polysaccharides (LBP) could elevate the exercise tolerance, reduce lipid per-oxidation and up-regulate antioxidant enzyme activity during exercise. This indicates that LBP is effective in preventing oxidative stress after exhaustive exercise.

## Figures and Tables

**Table 1.** Effects of *Lycium barbarum* polysaccharides (LBP) on body weight and endurance time of rats (mean ± SD, n = 12).

**Group**	**Body weight (g)**		**Endurance time (min)**
	**Before experiment**	**After experiment**	
NC	284.61 ± 28.46	434.54 ± 31.28	61.21 ± 4.22
LT	289.49 ± 21.37	427.39 ± 27.23	84.37 ± 6.28*
MT	292.34 ± 24.61	441.06 ± 22.84	88.94 ± 5.76*
HT	287.59 ± 30.25	429.17 ± 25.62	94.79 ± 5.94*

* *p* < 0.05 as compared with the normal control group (NC).

**Table 2.** Effects of LBP on super oxide dismutase (SOD) and glutathione peroxidase (GPX) levels of rats after exhaustive exercise (mean ± SD, n = 12).

**Group**	**SOD (U/mg·pro)**	**GPX(U/mg·pro)**
NC	101.48 ± 10.28	4.74 ± 1.25
LT	131.36 ± 9.41*	7.23 ± 0.96*
MT	148.69 ± 11.23*	10.37 ± 1.14*
HT	157.84 ± 12.65*	14.29 ± 1.29*

* *p* < 0.05 as compared with the normal control group (NC).

**Table 3.** Grouping of animals.

**Group**	**Number**	**Administration of animals**
Normal control (NC)	12	Rats were treated orally with physiological saline every day.
Low-dose LBP treated (LT)	12	Rats were treated orally with 100 mg/kg b.w. LBP every day.
Middle-dose LBP treated (MT)	12	Rats were treated orally with 200 mg/kg b.w. LBP every day.
High-dose LBP treated (HT)	12	Rats were treated orally with 400 mg/kg b.w. LBP every day.
